# Effect of High Hydrostatic Pressure on Human Trabecular Bone Regarding Cell Death and Matrix Integrity

**DOI:** 10.3389/fbioe.2021.730266

**Published:** 2021-08-12

**Authors:** Janine Waletzko-Hellwig, Christopher Pohl, Janik Riese, Michael Schlosser, Michael Dau, Nadja Engel, Armin Springer, Rainer Bader

**Affiliations:** ^1^ Department of Oral, Maxillofacial and Plastic Surgery, Rostock University Medical Center, Rostock, Germany; ^2^ Department of General Surgery, Visceral, Thoracic and Vascular Surgery, University Medical Center Greifswald, Greifswald, Germany; ^3^ Medical Biology and Electron Microscopy Center, Rostock University Medical Center, Rostock, Germany; ^4^ Biomechanics and Implant Technology Research Laboratory, Department of Orthopedics, Rostock University Medical Center, Rostock, Germany

**Keywords:** high hydrostatic pressure, bone replacement material, allograft, gel electophoresis, histology, cell dealth

## Abstract

The reconstruction of critical size bone defects is still clinically challenging. Even though the transplantation of autologous bone is used as gold standard, this therapy is accompanied by donor site morbidities as well as tissue limitations. The alternatively used allografts, which are devitalized due to thermal, chemical or physical processing, often lose their matrix integrity and have diminished biomechanical properties. High Hydrostatic Pressure (HHP) may represent a gentle alternative to already existing methods since HHP treated human osteoblasts undergo cell death and HHP treated bone cylinders maintain their mechanical properties. The aim of this study was to determine the biological effects caused by HHP treatment regarding protein/matrix integrity and type of cell death in trabecular bone cylinders. Therefore, different pressure protocols (250 and 300 MPa for 10, 20 and 30 min) and end point analysis such as quantification of DNA-fragmentation, gene expression, SDS-PAGE, FESEM analysis and histological staining were performed. While both protein and matrix integrity was preserved, molecular biological methods showed an apoptotic differentiation of cell death for lower pressures and shorter applications (250 MPa for 10 and 20 min) and necrotic differentiation for higher pressures and longer applications (300 MPa for 30 min). This study serves as a basis for further investigation as it shows that HHP successfully devitalizes trabecular bone cylinders.

## Introduction

Bone is one of the few human tissues which can regenerate and repair itself (Oryan et al., 2014). However, if the maximum critical defect size is exceeded e.g., due to tumor or trauma, self-regeneration is no longer possible (Nauth et al., 2018). In these cases, bone substitutes which should initiate and support osseous healing must be provided (Beaman et al., 2006). The requirements for such materials are diverse. In addition to osteoinductive, osteogenic and osteoconductive properties, biomechanical integrity for structural support is desirable as this is particularly important for load-bearing bone defects (Beaman et al., 2006; Parvizi and Kim, 2010). Since autologous bone combines all the above-mentioned positive aspects, it is still the gold standard used in reconstruction surgery (Sohn and Oh, 2019). Another important positive feature is the reduced risk of an immune response of the recipient and a low probability of transmitting diseases (Pape et al., 2010; Oryan et al., 2014). However, main disadvantages of using autografts are the limitation in graft quantity and availability. In addition, the occurrence of donor site morbidities as well as the increased physiological burden on patients due to two surgical interventions must be mentioned [1,6,7]. An alternative approach which solves problems of accessibility and complications at the harvest site during removal of autologous bone is the use of allografts. These are available in different forms e.g., as blocks or granules, so that an individual adaption to the needs of patients is possible (Shibuya and Jupiter, 2015; Baldwin et al., 2019). Various processing methods like gamma irradiation, freeze-drying, thermal and chemical processing or a combination are used with the aim of receiving a sterilized graft without any cellular remnants which may induce an immunological response (Mikhael et al., 2008; Cornu et al., 2011; Singh et al., 2016). However, the biological and biomechanical properties are often lost during processing. For example, freezing and freeze-drying lead to a lack of osteogenic properties, vascularity and immediate strength (Pape et al., 2010). Furthermore, it could be shown that gamma sterilization reduces both, stiffness and compressive strength compared to the untreated controls. Protein structures are also influenced negatively due to gamma irradiation of bone allografts: polypeptide chains were broken up whereby a reduction of biological and biomechanical properties could be observed in several studies (Açil et al., 2007; Nguyen et al., 2007).

A gentle alternative to already existing processing methods could be the treatment of bone specimens with high hydrostatic pressure (HHP). Several studies have shown that HHP is able to devitalize cells and various tissues (Masson et al., 2001; Funamoto et al., 2010; Waletzko et al., 2020). An inhibition of bacterial growth and an inactivation of viruses by means of HHP was also observed (Kingsley et al., 2002; Abe, 2007). At the same time, it could be proven that biomechanical properties regarding stiffness and stress at 15% strain are not influenced by HHP-treatment (Diehl et al., 2005; Waletzko‐hellwig et al., 2021). Preliminary work showed that osteoblasts, which are part of cancellous bone, react differently depending on the HHP application; while 100–150 MPa showed no effect, the cells acted primarily apoptotic to pressures from 250 to300 MPa, whereas a pressure of 450–500 MPa led to necrosis (Waletzko et al., 2020). Possibly, necrotic tissue components, will trigger strong immune responses in the recipient after transplantation and should, accordingly, be avoided. Thus, ideally the applied pressure should lead to apoptotic rather than necrotic behavior and therefore be selected carefully with regard to later medical application (Rivalain et al., 2010).

The purpose of this experimental study was to characterize the biological effects of HHP on human trabecular bone in terms of cell death and protein integrity. On basis of preliminary work, pressure applications of 250 and 300 MPa were chosen. In addition to a treatment period of 10 min, pressures were also applied for 20 and 30 min as a longer application could be necessary for complete devitalization due to the three-dimensional tissue structure of bone blocks. DNA fragmentation caused by HHP was analysed by gel electrophoresis. Furthermore, the occurrence of apoptosis-specific genes was determined at gene expression level. In addition, an SDS-PAGE was used to examine the protein integrity. Histological analyses were performed to assess whether HHP causes a homogeneous distribution of apoptosis in the bone blocks. Finally, field emission scanning electron microscopy (FESEM) was used to estimate possible superficial damage to the bone matrix after HHP-treatment.

## Materials and Methods

**FIGURE 1:**
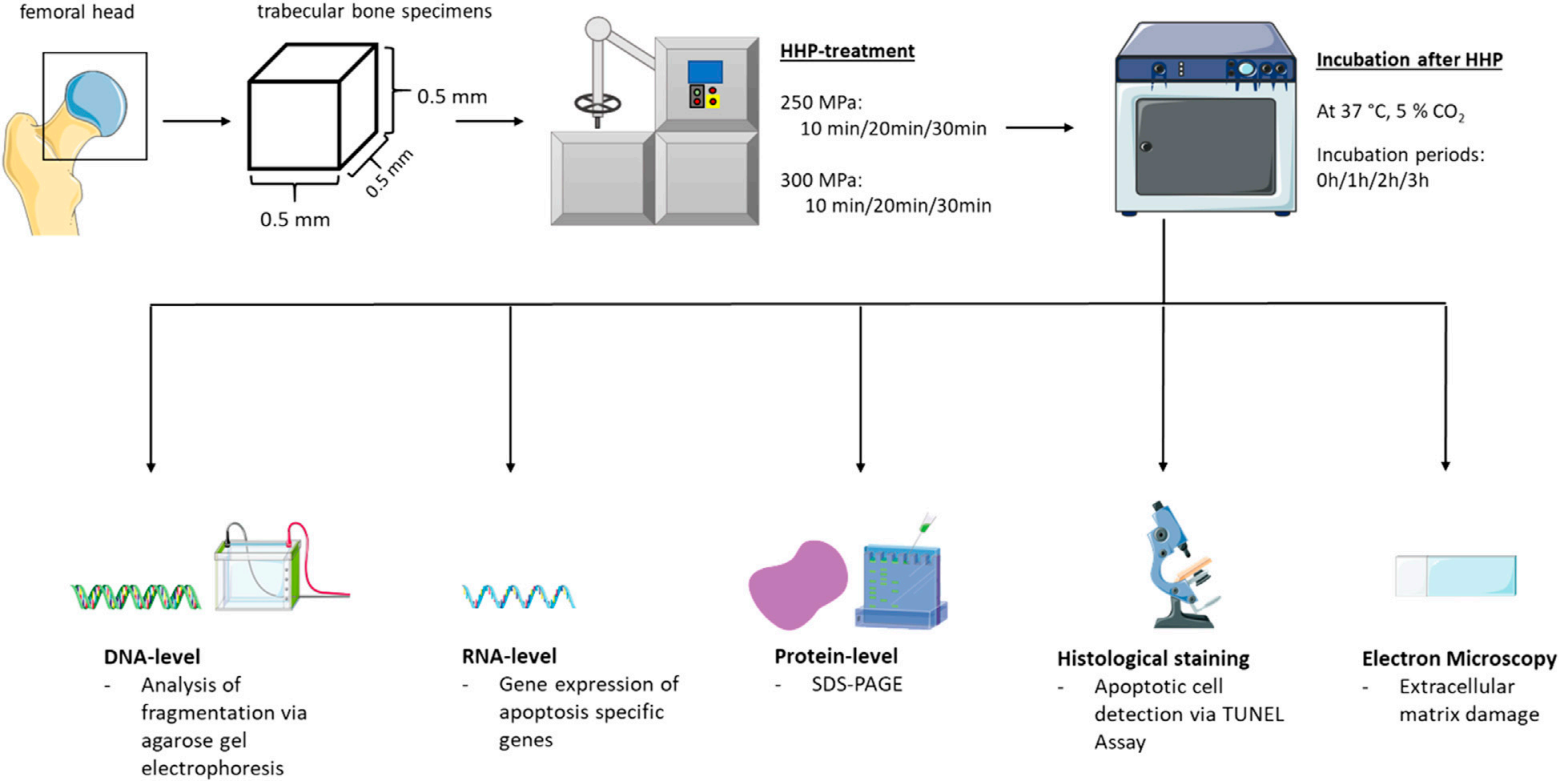
Schematic overview of the methods used.

An overview of the methods used to answer the present research question is shown in Figure 1. The extraction of bone blocks, the HHP-treatment and the analyses on DNA-/RNA- and protein-level as well as the imaging analyses are explained in more detail below.

### Sample Preparation and High Hydrostatic Pressure Treatment

Trabecular bone specimens were harvested from femoral heads of patients undergoing a total hip joint replacement. Before surgery, patient consent and ethical approval from the ethics committee of the University Rostock, Germany were obtained (ethics approval number A 2010–0010). Specimens were stored at −20°C until further preparation. Prior to sample manufacturing, femoral heads were rinsed with sterile phosphate-buffered saline (PBS) (Sigma Aldrich, Munich, Germany), supplemented with 1% penicillin/streptomycin (Sigma Aldrich, Munich, Germany). Afterwards, the cancellous parts of the tissues were sawed into bone blocks of a size of 125 mm^3^ using a diamond coated bone saw (EXAKT Advanced Technologies GmbH, Norderstedt, Germany). For High Hydrostatic Pressure (HHP) -treatment, bone blocks were then transferred into 2 ml CryoTubes (ThermoFisher Scientific, Waltham, MA, United States) filled with Dulbecco’s modified Eagle’s medium (DMEM) (PAN-Biotech, Aidenbach, Germany) supplemented with 10% fetal calf serum (FCS, PAN-Biotech), 1% amphotericin B, 1% penicillin/streptomycin and 1% HEPES buffer (all: Sigma-Aldrich, Munich, Germany). The samples were treated with different pressure protocols (250 MPa for 10, 20 and 30 min and 300 MPa for 10, 20 and 30 min by means of the 206,797–Druckprüfanlage 6,000 bar, Dustec Hochdrucktechnologie, Wismar, Germany) at a constant temperature of 30°C. The control groups were prepared in the same way as the HHP treated groups but received no HHP treatment. During HHP application, untreated controls were incubated at 30°C. Afterwards, samples were incubated for 0, 1, 2 and 3 h at standard culture conditions (37°C, 5% CO_2_). These incubation periods after HHP application were chosen to track time courses of apoptotic signaling cascades, since previous studies showed that DNA fragmentation could only be detected 2 h after apoptosis inducing treatments (Hug, 2000). After the incubation period, specimens were stored according to their further use; bone blocks, from which proteins were to be isolated, were shock frozen in liquid nitrogen immediately after the incubation period had elapsed. Samples for subsequent DNA/RNA-isolation were stored at -80°C. Those specimens which were intended for histological analyses or field emission scanning electron microscopy (FESEM) were stored at room temperature and fixed with Formafix^©^ (histological analysis) (PathoMed. Logistik GmbH, Viersen, Germany) or with fixation buffer (FESEM) (1% paraformaldehyde, 2.5% glutaraldehyde, 0.1 M sodium phosphate buffer, pH 7.3).

### DNA Isolation From Trabecular Bone Blocks and Gel Electrophoresis

Frozen bone specimens were thawed at 37°C using a water bath. Afterwards the blocks were digested overnight, using 1 ml collagenase A (Roche Diagnostics GmbH, Mannheim, Germany) per bone block. Non-digestible components were removed and the suspension was centrifuged at 1,000x g for 5 min. After removal of the supernatant, the pellet was resuspended with 500 µL isolation buffer (Tris-EDTA-buffer, pH 7.5, 2% SDS, 2% Triton X-100). After centrifugation at 12,000x g for 5 min and 4°C the supernatant was transferred into a 1.5 ml reaction tube following the addition of 750 µL ice cold 100% ethanol. The samples were centrifuged at 12,000x g for 5 min at 4°C, the supernatant was discarded and the precipitated DNA was dissolved in 50 µL TE-buffer (pH 7.5). DNA concentration was measured using a Tecan Reader Infinite^®^ 200 Pro microplate reader and NanoQuant™ Plate (Tecan Trading AG, Maennedorf, Schwitzerland) with TE-buffer as blank.

DNA-fragmentation was analysed by performing a gel electrophoresis using a 1.7% agarose gel with 0.01% SYBR™Safe DNA gel stain (ThermoFisher Scientific, Waltham, MA, United States). Samples were mixed with TriTrack loading Dye (ThermoFisher Scientific) in a 1:5 dilution. A Gene Ruler 100 bp DNA ladder (ThermoFisher Scientific) was used as a marker and was prepared according to the user manual’s loading protocol. Per sample 500 ng DNA was loaded on the gel and a voltage of 80 V was applied till the sample left the gel wells. Afterwards a voltage of 120 V was applied for at least 20–30 min. The imaging was performed using the ChemiDoc™ Molecular Imager (BioRad Laboratories, Hercules, CA, United States) with the Image Lab Software (BioRad Laboratories) using the application “Nucleic Acid Gels”.

### RNA Isolation From Trabecular Bone Blocks and Reverse Transcription Polymerase Chain Reaction

For RNA isolation from trabecular bone blocks, samples were digested overnight and afterwards centrifuged as described above. The received pellet was resuspended with 1 ml TRIzol Reagent™ (ThermoFisher Scientific) and incubated for 5 min at room temperature. 200 µL chloroform were added and the samples were again incubated for 2–3 min at room temperature following a centrifugation step at 12,000 x g for 15 min at 4°C. The aqueous phase, which contained the RNA, was removed and 1 ml of ice-cold 100% ethanol was added. After an incubation period of up to 3 h at -20°C, samples were centrifuged at 12,000 x g for 10 min at 4°C. The supernatant was discarded and the pellet was washed with 500 µL ice-cold 70% ethanol following a centrifugation at 13,500 rpm for 10 min at 4°C. The RNA was precipitated again using 500 µL ice-cold 100% ethanol and centrifuged under the same conditions as described before. The supernatant was discarded and the pellet was dried at room temperature. Afterwards, the RNA was resuspended in 100 µL TE-buffer and RNA concentration was measured analogous to DNA concentration measurement.

For reverse transcription polymerase chain reaction (RT-PCR), 100 ng RNA was used. The RT-PCR was performed according to manufacturer’s recommendations of the Revert Aid First Strand cDNA Synthesis Kit (ThermoFisher). The template RNA and the master mix containing Oligo (dT)_18_ primer, reaction buffer, RiboLock RNase Inhibitor, dNTP Mix and RevertAid M-MulV RT, were mixed and the PCR protocol was carried out as follows: 1 h at 37°C, 5 min at 70°C. Afterwards, samples were stored at −20°C for further use.

#### Transcript Expression Analysis of Apoptosis-specific Primers

The regulation of apoptosis-specific genes was evaluated at the gene expression level by the detection of the Fas Cell Surface Death Receptor (*fas*) and Caspase-8 (*casp-8*) (both: Sigmal-Aldrich, Darmstadt, Germany). The primers are listed in Table 1.

**TABLE 1:** Primer sequences for PCR.

Primer	Sequences (5–3′)
Fas Cell Surface	*fwd:* TGA​CCC​TTG​CAC​CAA​ATG​TGA
Death Receptor (Fas)	*rev:* AGA​CAA​AGC​CAC​CCC​AAG​TT
Caspase-8 (Casp-8)	*fwd:* TCA​CAG​GTT​CTC​CTC​CTT​TTA​TCT​T
	*rev:* GCA​GGA​GAA​TAT​AAT​CCG​CTC​CA

A master mix was prepared for each gene and sample, containing 1 µL forward primer, 1 µL reverse primer, 7 µL H_2_O and 10 µL DreamTaq Green PCR Master Mix (ThermoFisher). Next, 19 µL Master mix was added to 1 µL cDNA and PCR was performed (My Cycler™, Biorad) applying the following protocol: 1 cycle of 95°C (5 min), 40 cycles of 95°C (30s), 58°C (30s), 72°C (1 min) and 1 cycle of 72°C (5 min).

Samples were then loaded on a 1% agarose gel with 0.01% SYBR™Safe DNA gel stain. Gel electrophoresis and subsequent imaging was carried out as described above.

### Protein Isolation and From Trabecular Bone Blocks and Protein Quantification

All steps during protein isolation were performed on ice. After a gentle defrosting of the specimens, 600 µL of ice-cold Pierce™ RIPA buffer (ThermoFisher Scientific) was added, containing 0.02% animal component free protease inhibitor cocktail (Sigma-Aldrich, Munich, Germany). After an incubation period of 10 min, specimens were homogenized with an ultrasonic homogenizer UP100H (Hielscher Ultrasonics GmbH, Teltow, Germany) with an amplitude of 80% and a cycle of 0.5 for 90s. Samples were cooled again on ice for 5 min. Larger tissue debris was then removed and the lysate was centrifuged at 12,500x g for 15 min. The supernatants were collected for measurement of protein concentration and SDS-Page.

Determination of protein concentration was performed via Bradford assay using Roti^®^Quant solution (Carl Roth GmbH & Co. KG, Karlsruhe, Germany). Thus, 499 µl solution and 1 µl protein solution were incubated for 10–15 min and the absorption at 595 nm was measured. Using a BSA standard curve, the protein concentration per sample was calculated.

#### SDS-PAGE

In preparation for SDS-PAGE, protein samples were mixed with an equal volume of Lämmli Buffer (Sigma-Aldrich) and incubated at 95°C for 5 min. Afterwards, samples were put on ice immediately. The SDS-PAGE was performed using a Mini-PROTEAN TGX Stain-Free Any kD Precast Gel (BioRad Laboratories). Per lane 10 µg total protein was loaded on the gel. In order to determine the band size, the Precision Plus Protein™ Dual Color Standard (BioRad Laboratories) was carried along with every SDS-PAGE. First, a voltage of 60 V was applied until the proteins had left the gel pockets, then the voltage was increased to 120 V for at least 30 min. The imaging was performed as already described above, now using the application “Protein Gels”.

### Analysis of Surface Structure of Bone Blocks by Field Emission Scanning Electron Microscopy (FESEM)

The bone blocks stored in fixation buffer were prepared for FESEM analysis as described in (Waletzko et al., 2020). Images were taken with a field emission scanning electron microscope (MERLIN VP Compact, Carl Zeiss, Oberkochen Germany), using a HE-SE2 (High Efficiently Secondary Electron 2) detector, an accelerating voltage of 5.0 kV and a working distance of 5.2 mm.

### Histological Analysis

Before decalcification of the bone platelets a fixation step was performed using a buffered formalin solution. Therefore, 0.06 M KH_2_PO_4_ (Sulpeco, Bellefonte, PA, United States) was combined with 0.08 M Na_2_HPO_4_ (VWR, Darmstadt, Germany) and both were diluted in 860 ml Aqua dest. Finally, 140 ml formalin (37% WT; Sigma Aldrich, St. Lois, MO, United States) was added to the solution. The samples were treated with the fixation medium for 24 h at room temperature. After fixation, the tissue was washed for 2 h with tap water. EDTA-decalcification solution was produced by combining 14 g EDTA (Acid-form; Sigma Aldrich, St. Lois, MO, United States) with 9 ml ammonia solution (Merck, Darmstadt, Germany) and 76 ml Aqua dest. During incubation, the solution was kept in constant motion via a stirring magnet to reduce saturation effects. At high turbidity medium was renewed. Decalcification time differed from 1 to 3 weeks, depending on the size of the bone plate. To verify sufficient decalcification, a needle test was performed. For sectioning, tissue samples were placed in embedding medium consisting of a 1:1 dilution of TissueTek (Sakura, Osaka, Japan) with 1xPBS and shock frozen in liquid nitrogen in square Peel-A-Way containers (TED- PELLA, INC., Redding, Canada). Then, 10 µm thin cryosections were created with a CM 3050 S Cryotome (Leica, Nussloch, Germany). Sections were fixed with 4% paraformaldehyde for 10 min at room temperature, stained with an *in situ* cell death detection kit (Roche, Basel, Switzerland) and counterstained with DAPI (Molecular Probes, Eugene, OR, United States). Stained sections were stored at 4°C for at least 24 h before microscopy. All images were captured on the same day at a constant exposure time (TUNEL 1/50s, DAPI 1/5s) and magnification (20x). Afterwards images were merged to create whole sample images with a high resolution. From single images, apoptotic cells were identified by positive TUNEL staining and quantified using QuPath (Bankhead et al., 2017). Apoptotic cell density was calculated and transferred into a matrix to maintain spatial coherence. The data was subsequently normalized using a two-dimensional Gaussian distribution. The high affinity of bone tissue to DAPI was used to classify trabecular structures utilizing Otsu thresholding, thereby creating a Boolean image. The Boolean image was then combined with the normalized data matrix to visualize spatial apoptotic density distribution throughout the sample. Version 1 of this script as used in this publication was uploaded onto github, accessible under: https://github.com/ChrisPohl/Apoptotic-density-mapping.

### Evaluation of Gel Electrophoresis and Statistical Analysis

Evaluation of the agarose gels was done using the Image Lab Software (BioRad Laboratories). First of all, each picture was inverted and lanes and bands were detected using the automatic lane and band finder. If any problems arose here (e.g., too many lanes were detected, band detection was not right), they were corrected manually. Using the “Volume Tools”, the size of the bands could be determined and thus the number of pixels of each band was calculated. To quantify DNA fragmentation, all bands per lane were detected and categorized according to the following: < 200 bp, 200–500 bp, > 500 bp. For each group, the numbers of pixels were summarized and the groups were then set in relation to each other to determine the distribution over all groups.

To evaluate gene expression, lanes and bands of the gels were detected as described before. The number of pixels for each band was calculated with the “Volume Tools” of the software. Then, the relative gene expression related to the control group was calculated using the following equation:

$$gene\;expression\left[\%\right]=\frac{number\;of\;pixels\;\left(treated\right)}{number\;of\;pixels\;\left(control\right)}.$$

The results are presented as means with interquartile ranges (25–75%) and whiskers. Statistical analysis was done by two-way-ANOVA tests using GraphPad Prism Version 7 (GraphPad Software, San Diego, CA, United States), Bonferroni’s multiple comparison test was applied as a post hoc analysis. *p*-values ≤ 0.05 were deemed as significant.

## Results

### Characterization of DNA After HHP-Treatment

**FIGURE 2:**
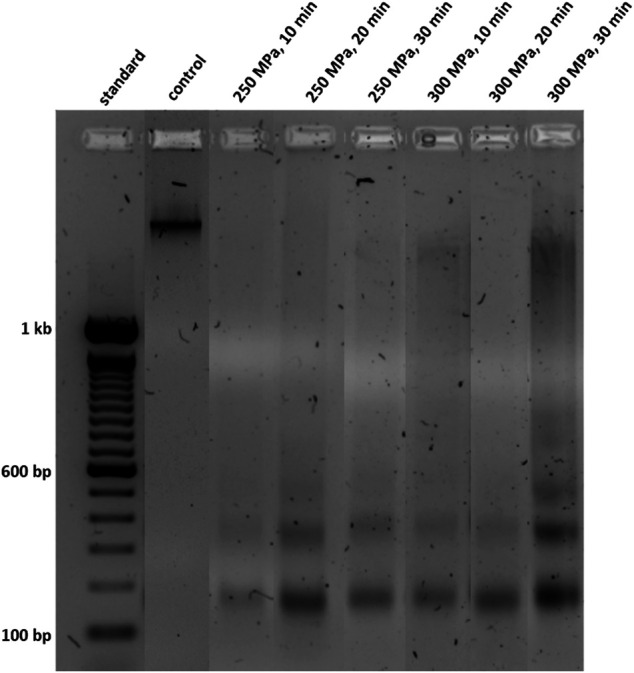
Oligonucleosomal size fragmentation of DNA isolated from human trabecular bone. Shown samples were incubated for 1 h after HHP-treatment. DNA was separated using a 1.7% agarose gel and a voltage of 120 V.

To investigate the effect of HHP on trabecular bone, the first step was to analyse the DNA degradation by gel electrophoresis. Figure 2 shows an example of a gel which contains a control sample as well as different treated samples. In the untreated sample, only one large fragment of >1 kb could be detected while the HHP-treated samples showed fragments of all sizes especially those below 200 bp. Furthermore, it is noticeable that some treatments, such as lane 6 (300 MPa, 10 min) and 8 (300 MPa, 30 min), have led to a smearing of DNA in the gel.

**FIGURE 3:**
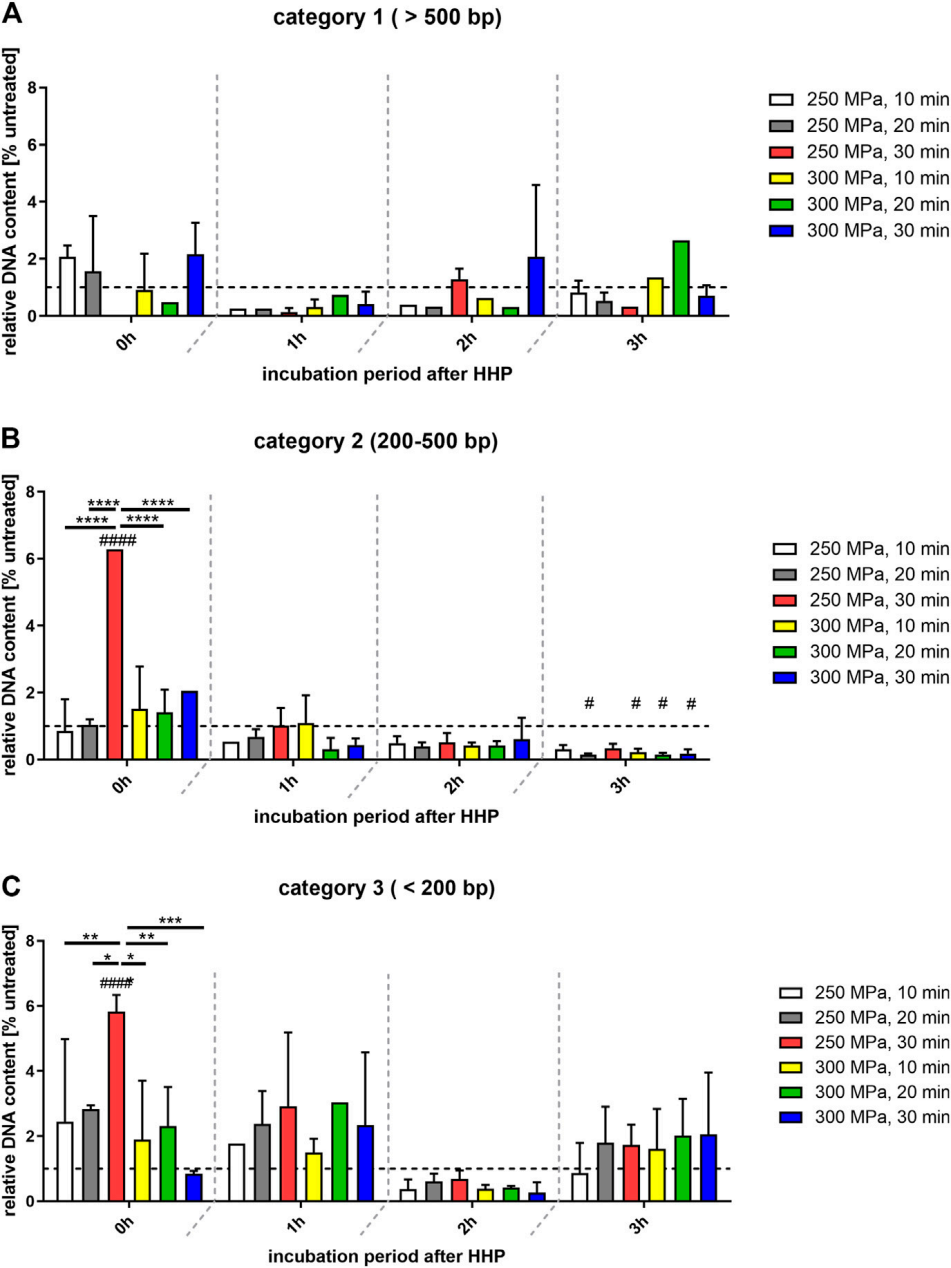
Relative quantification of DNA-content of different fragment categories related to the control group (100%; shown as broken line). **(A)** Category 1: >500 bp; **(B)** category 2: 200–500 bp; **(C)** category 3: < 200 bp. Data shown as mean ± SD of 7 independent donors. Statistical analysis was performed using two-way-ANOVA with Bonferroni’s multiple comparison test as post hoc test. Significantly different from untreated control: #*p* ≤ 0.05; ####*p* ≤ 0.0001. Black bars show significant differences between the different treatments within the same incubation period. *p ≤ 0.05; **p ≤ 0.01; ***p ≤ 0.001; ****p ≤ 0.0001.

For further quantification, the detected bands were categorized into three different groups (<200 bp, 200–500 bp, > 500 bp) and a densitometric analysis was performed. The results are shown in Figure 3. For a better visualization, significant differences between the treatments within an incubation period are depicted in the graphs of Figure 3. Significant differences over time are shown in Table 2.

**TABLE 2:** Overview of the statistically significant results of the DNA-fragment analysis over time.

Category	HHP-treatment	Analysed groups	*p*-value
200–500 bp	250 MPa, 20 min	0 h vs. 3 h	0.0118
	250 MPa, 30 min	0 h vs. 1 h	<0.0001
	250 MPa, 30 min	0 h vs. 2 h	<0.0001
	250 MPa, 30 min	0 h vs. 3 h	<0.0001
	250 MPa, 30 min	1 h vs. 3 h	0.0367
	300 MPa, 10 min	0 h vs. 2 h	0.0205
	300 MPa, 10 min	0 h vs. 3 h	0.0003
	300 MPa, 10 min	1 h vs. 3 h	0.0069
	300 MPa, 20 min	0 h vs. 1 h	0.0051
	300 MPa, 20 min	0 h vs. 2 h	0.0019
	300 MPa, 20 min	0 h vs. 3 h	0.0002
	300 MPa, 30 min	0 h vs. 1 h	0.0035
	300 MPa, 30 min	0 h vs. 2 h	0.0108
	300 MPa, 30 min	0 h vs. 3 h	0.0002
<200 bp	250 MPa, 20 min	0 h vs. 2 h	0.0455
	250 MPa, 30 min	0 h vs. 1 h	0.0047
	250 MPa, 30 min	0 h vs. 2 h	<0.0001
	250 MPa, 30 min	0 h vs. 3 h	<0.0001
	250 MPa, 30 min	1 h vs. 2 h	0.0436

Figure 3A shows that the DNA content of the treated samples detected in category 1 (>500 bp) was predominantly lower than that of the control group. Comparing the treated groups with each other, no significant differences were found here either. Thus, it can be seen that more rather large oligonucleosomal fragments occur in the control group than in the treated groups. In case of a HHP application of 300 MPa for 30 min it could be seen, that at incubation periods of 0 and 2 h the DNA content >500 bp was higher than those of the control. This was probably caused by the smear observed in Figure 1, which is assigned to category 1 by the evaluation method.

In case of category 2 (200–500 bp, Figure 3B), the distribution of DNA content across the treatment groups is not quite as even. At the time points of 1, 2 and 3 h after HHP, all treated groups are below or at the level of the values of the control group. However, at the time point 0 h after HHP the number of base pairs that can be allocated to this category increased compared to the control. This is also reflected in the significant differences within the individual treatment groups at different incubation times (Table 2).

In category 3 (<200 bp; Figure 3C), the highest increase was observed after 0 and 1 h incubation after HHP compared to the control group. This enormous increase is significant both over the incubation periods after HHP and over the various pressure parameters for the group “250 MPa, 30 min”. Furthermore, for the applied pressure of 250 MPa, it can be observed that in case of 0 and 1 h incubation after HHP, the DNA content of the bands <200 bp increases depending on the applied pressure time; the longer the HHP-treatment time, the higher the DNA content of bands <200 bp. After a decrease in DNA content of all treated groups below the level of the control 2 h after HHP, at 3 h after HHP another increase was observed in category 3 (Figure 3C).

### Gene Expression of Apoptosis Specific Genes in Human Trabecular Bone Specimens After HHP Treatment

In order to better assess the effect of HHP on trabecular bone blocks, gene expression analyses were carried out in the next step. Since it is known from literature that pressures below 300 MPa primarily lead to apoptosis, genes that are part of the apoptotic signaling cascade were selected for gene expression analyses.

**FIGURE 4:**
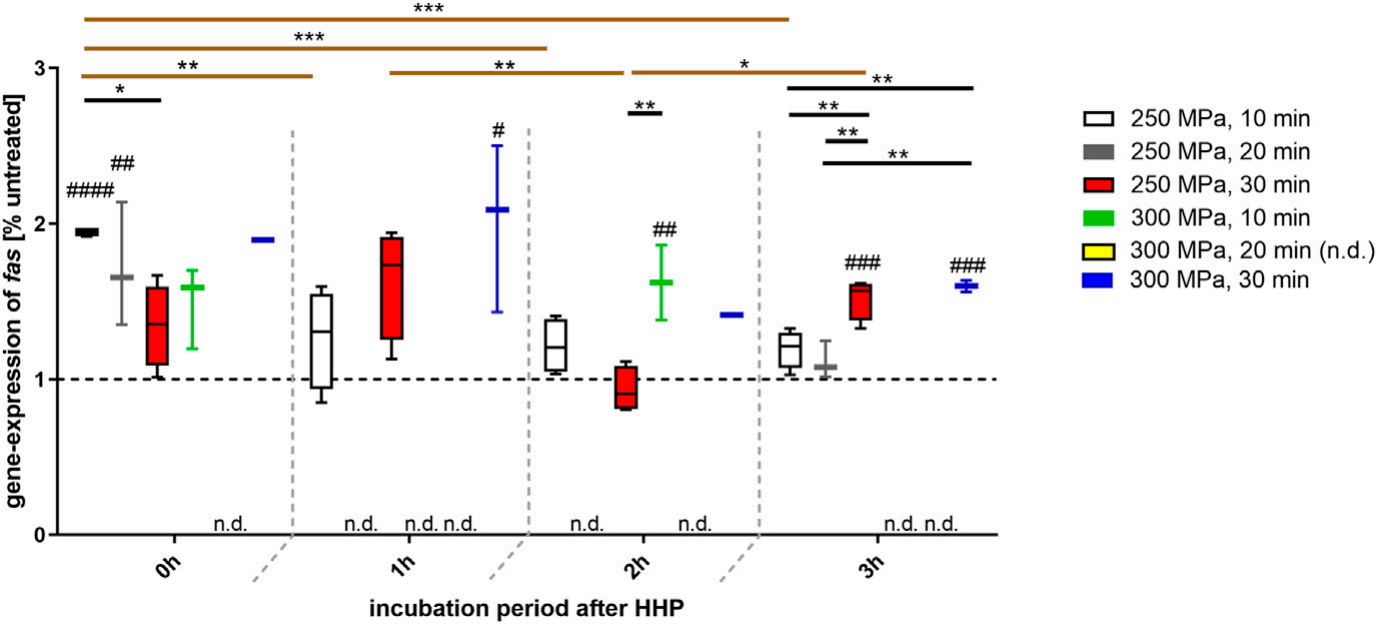
Gene expression of *fas* in human trabecular bone following HHP-treatment. Data are depicted as box plots with means and interquartile ranges from 25 to 75%. Gene expression is shown as percentage of untreated tissue (broken line at 1). Statistical Analysis was performed using one-way-ANOVA and two-way-ANOVA (*n* = 4) with Bonferroni’s multiple comparison test as post hoc test. Significantly different from untreated control: #*p* ≤ 0.05; ##*p* ≤ 0.01; ###*p* ≤ 0.001; ####*p* ≤ 0.0001. Significant differences between HHP-treatments: *p ≤ 0.05; **p ≤ 0.01; ***p ≤ 0.001. Black lines show significant differences between the different treatments within the same incubation period, brown lines show significant differences between the same treatments at different points of incubation time. Abbreviations: n.d. = not detectable.

For all depicted groups, an overexpression of *fas* could be detected, whereby this was far above the level of the control, especially at incubation time point 0 h after HHP-treatment (Figure 4). Regardless of the incubation period after HHP-treatment, some HHP applications, e.g., 300 MPa for 30 min, have led to a constant overexpression of *fas*. However, it should be noted that this can only be regarded as light tendency in case of 0 and 2 h after HHP treatment, since the expression of *fas* could only be detected in one of four donors and is therefore not statistically significant. In contrast, a continuous and significant decrease in *fas* expression over the incubation period was observed, for example with a HHP application of 250 MPa for 10 min. However, this decrease did not undercut the control level. In contrast, longer HHP applications (in this case 250 MPa, 30 min) led to a fluctuation in gene expression; while a higher expression was observed at incubation times 0 and 1 h after HHP, it dropped to the control level at incubation time 2 h and was clearly above the control level again at incubation of 3 h after HHP.

**FIGURE 5:**
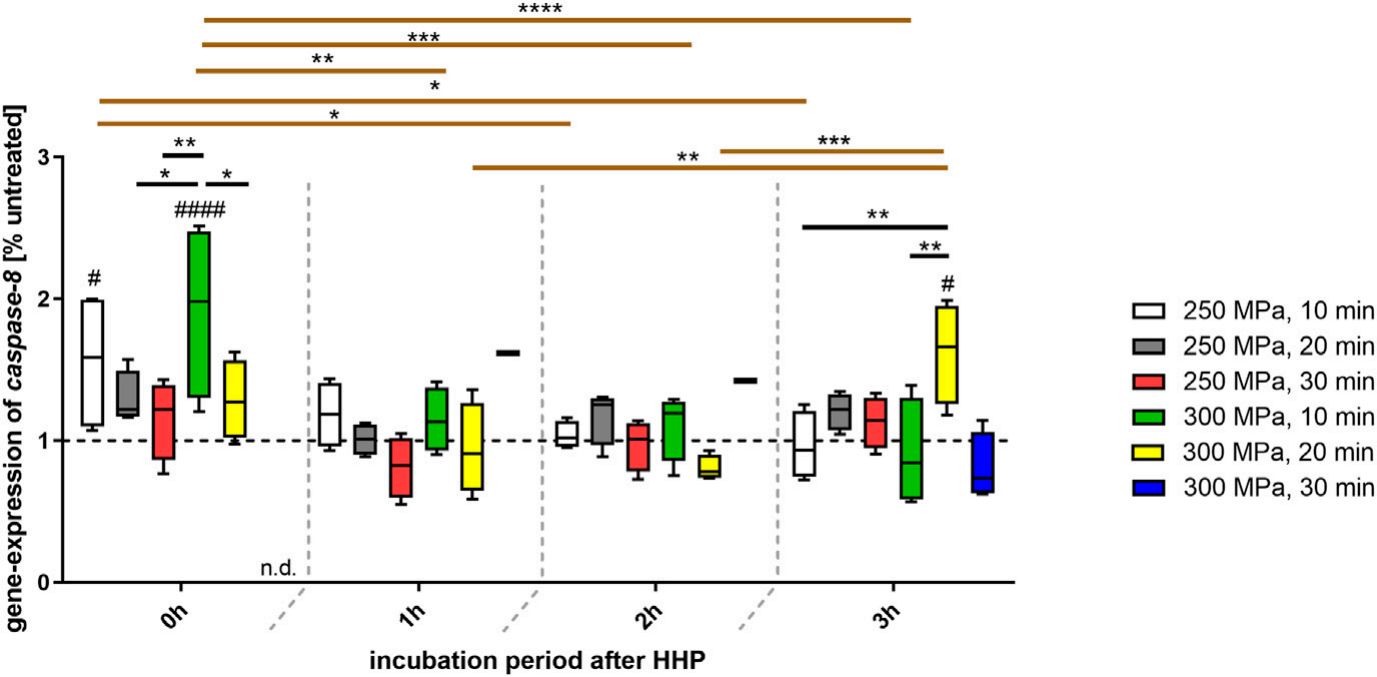
Gene expression of *caspase-8* in human trabecular bone following HHP-treatment. Data are depicted as box plots with means and interquartile ranges from 25 to 75%. Gene expression is shown as percentage of untreated tissue (broken line at 1). Statistical Analysis was performed using two-way-ANOVA (*n* = 4) with Bonferroni’s multiple comparison test as post hoc test. Significantly different from untreated control: #*p* ≤ 0.05; ####*p* ≤ 0.0001. Significant differences between HHP-treatments: *p ≤ 0.05; **p ≤ 0.01; ***p ≤ 0.001; ****p ≤ 0.0001. Black lines show significant differences between the different treatments within the same incubation period, brown lines show significant differences between the same treatment at different points of incubation time. Abbreviations: n.d. = not detectable.

Even more clearly than the previously observed *fas* gene expression, the analysis of *caspase-8* gene expression showed a progression in dependence of the incubation periods following HHP (Figure 5). While a HHP application of both 250 and 300 MPa for 10 min resulted in an overexpression of *caspase-8*, the expression decreased steadily over time up to the level of the control at 3 h after HHP-treatment. In case of a prolonged HHP application of 20 and 30 min, a fluctuation could be seen. Whereas a continuous decrease in expression was observed from 0 to 1 h incubation after HHP, *caspase-8* expression tended to rise above the control line again at the time point 2 h after HHP. This level was also maintained at the time of 3 h incubation. This behavior could be identified for a treatment of 250 MPa for 20 as well as 30 min HHP-treatment. However, while the treatment of 300 MPa for 20 min also showed this time-dependent tendency, a renewed increase of *caspase-8*-expression could only be observed after 3 h of incubation.

### Characterization of Protein Integrity via SDS-PAGE

SDS-PAGE (sodium dodecyl sulfate polyacrylamide gel electrophoresis) was used as a method for assessing protein integrity after HHP-treatment.

**FIGURE 6:**
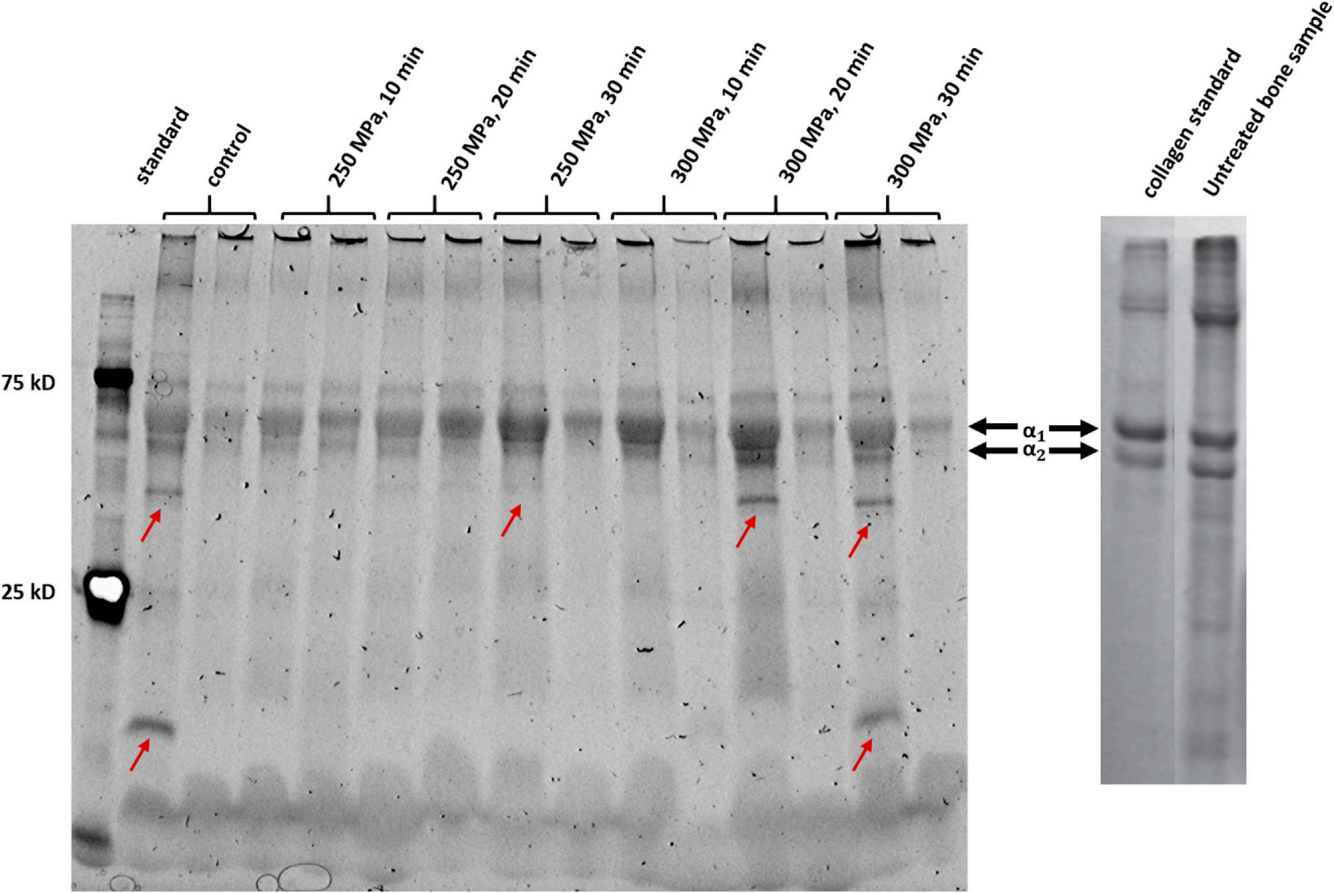
Left picture: SDS-PAGE of HHP-treated and untreated bone samples. The Precision Plus Dual Color Standard (BioRad Laboratories) with the specific bands at 25 kD and 75 kD was applied in the first lane from the left. Red arrows show conspicuous bands that occur only sporadically in some samples. Picture shows samples, which were shock-frozen immediately after HHP-treatment (incubation period 0 h). Right picture: Reference SDS-PAGE of an untreated bone sample, with a collagen standard for bone (adapted from Açil et al., 2007) and self-edited. Prominent bands which can also be seen in the reference gel were identified as alpha-1 and alpha-2 chains of collagen 1.

**FIGURE 7:**
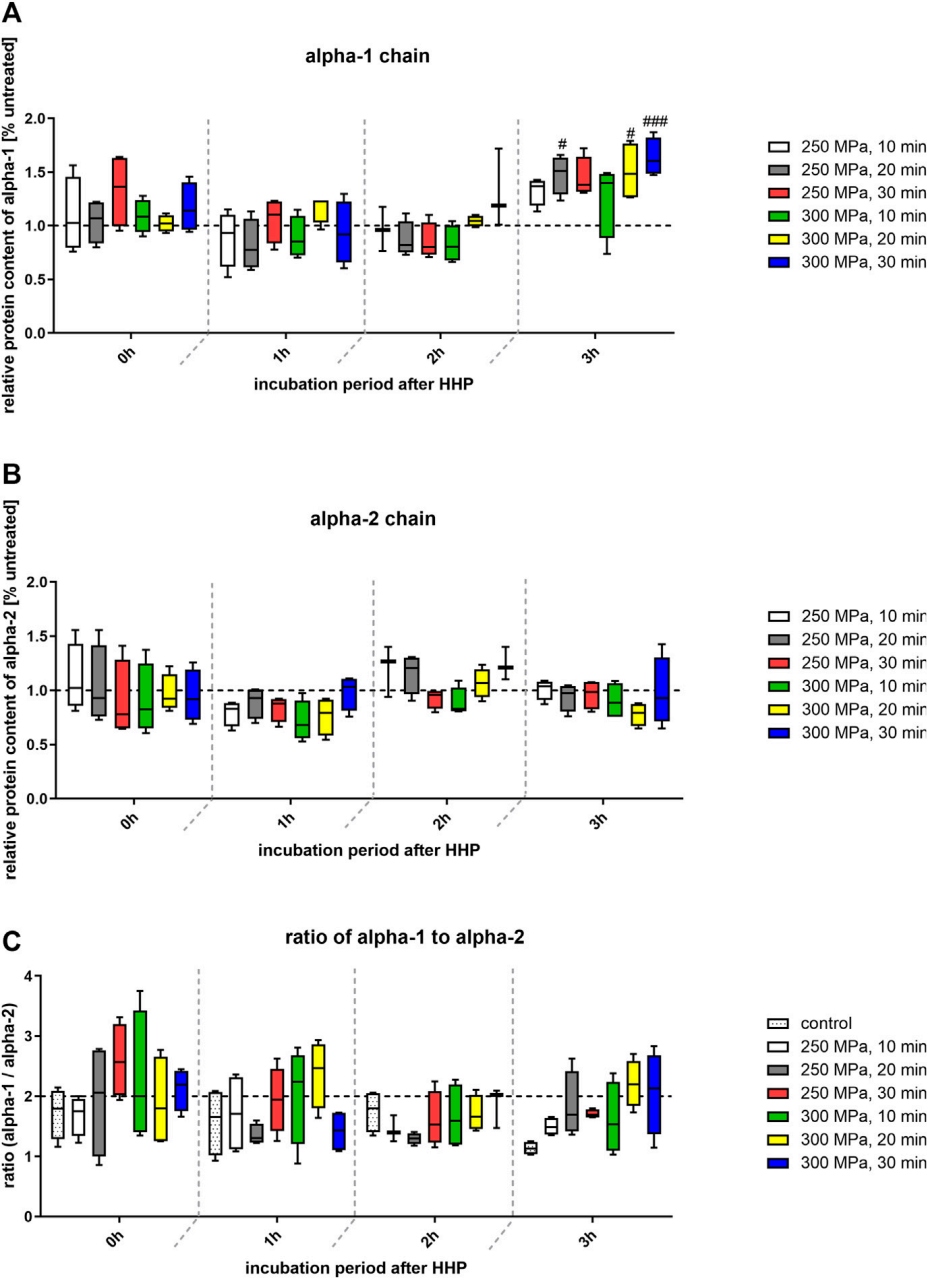
**(A)** Quantification of band intensity of alpha-1 chains from SDS-PAGE relative to intensity of control bands (dotted line at y = 1). **(B)** Quantification of band intensity of alpha-2 chains from SDS-PAGE relative to intensity of control bands (dotted line at y = 1). **(C)** Ratio of pixels of alpha-1 and alpha-2 bands. Dotted line at y = 2 shows the expected value of calculation. Statistical analysis was performed using two-way-ANOVA (*n* = 4) with Bonferroni’s multiple comparison test as post hoc test. Significant differences to untreated control: #*p* ≤ 0.05; ####*p* ≤ 0.0001.

Figure 6 shows an SDS-PAGE of HHP-treated and untreated trabecular bone samples. The detected bands from both, control and treated samples, are clearly visible. Furthermore, no smear could be observed. In all samples, a protein band pattern could be identified in the upper third of the gel. One protein band with a high molecular weight and two smaller bands of a lower molecular weight (slightly below 75 kD) which are close to each other could be found. This pattern also occurs in the reference gel from literature for both, the sample and the collagen 1 standard (Açil et al., 2007). Structurally, collagen 1 is a triple-helix composed of two alpha-1 chains and one alpha-2 chain, which differ slightly in their molecular mass (Fan et al., 2012). Considering this, the observed dominant bands could be identified as alpha-1 chains and alpha-2 chains. As described before, the protein content of alpha-1 and alpha-2 chains was quantitatively evaluated by densiometric analysis (Figures 7A,B). In addition, the ratio of intensity of the detected alpha-1 and alpha-2 bands was calculated (Figure 7C).

The relative protein content of alpha-1 chains compared to the control group was located at a similar level over the incubation period. Only after an incubation period of 3 hours after HHP a significant increase in the protein content and thus in the band strength could be detected in some treated groups compared to the control (Figure 7A). In case of the alpha-2 chain quantification, no significant differences could be detected, neither between the treatment groups, nor over time. As described above, collagen 1 is composed of two alpha-1 chains and one alpha-2 chain, so the chains exist in a 2:1 ratio. Based on this theoretical distribution, the quotient should be two. To verify whether this ratio also occurs in the presented samples, the densiometric results of the alpha-1 bands were divided by the densiometric results of the alpha-2 bands. As can be seen in Figure 7C, all calculated values are approximately at the level of 2. Furthermore, no significant differences between the groups could be detected.

### Bone Surface Structure Analysis

Field Emission Scanning Electron Microscopy was carried out following HHP-treatment to determine the surface matrix integrity of trabecular bone blocks.

**FIGURE 8:**
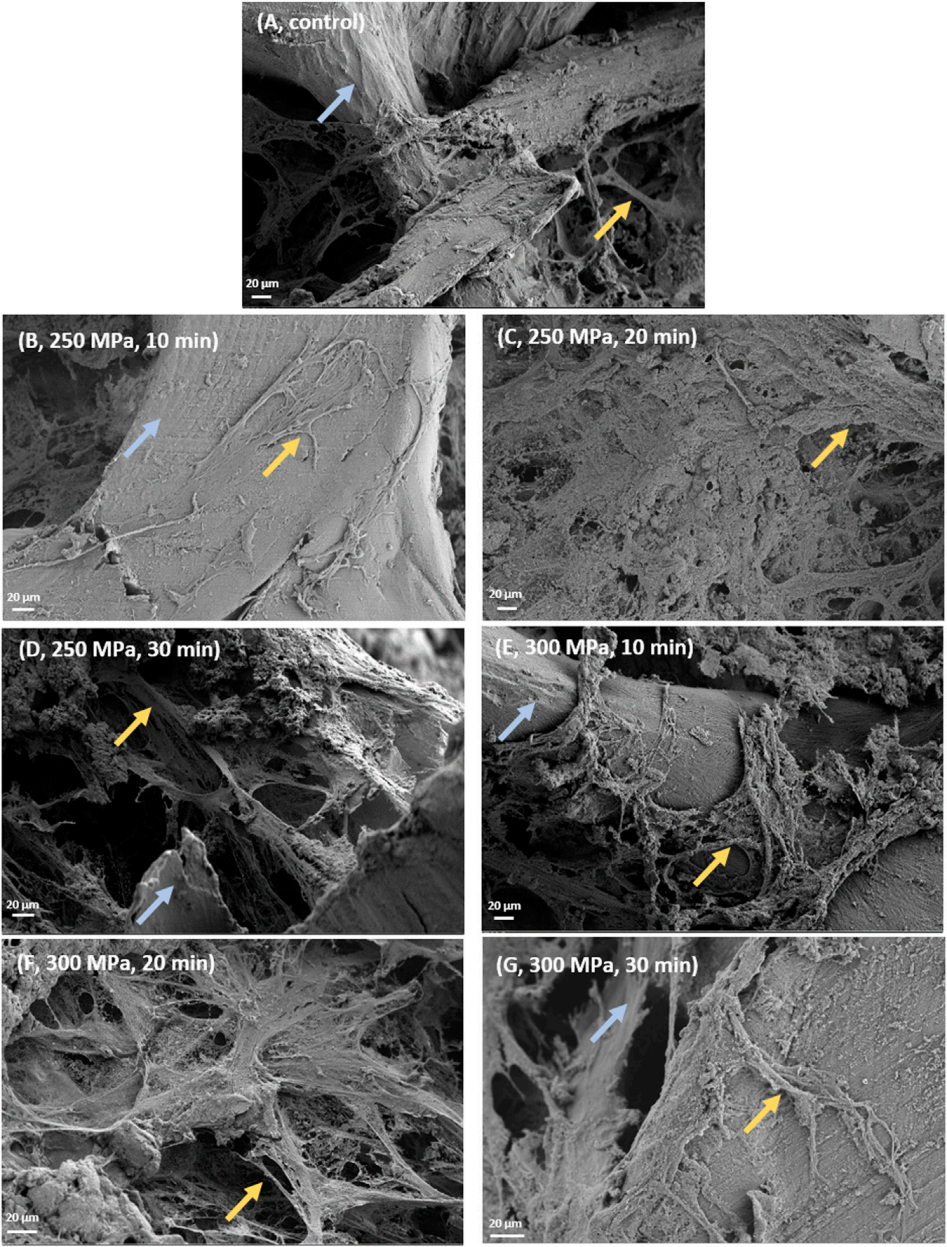
Scanning electron microscopy of human trabecular bone following HHP-treatment. **(A)** untreated control, magnification ×239; **(B)** 250 MPa, 10 min, magnification ×283; **(C)** 300 MPa, 10 min, magnification ×246; **(D)** 250 MPa, 20 min, magnification ×237; **(E)** 300 MPa, 20 min, magnification ×282; **(F)** 250 MPa, 30 min, magnification ×280; **(G)** 300 MPa, 30 min, magnification ×415. Blue arrows tag trabeculae, yellow arrows highlight cellular structures.

Figure 8. shows an overview of electron microscopic images of trabecular bone samples. Cell components are highlighted with yellow arrows and could be identified in all images. Cells were arranged in elongated filaments across the trabeculae within all samples. Also, the trabeculae could be clearly detected in almost all images (indicated with blue arrows). Apart from the appearance of cell components, no differences can be observed between the structures of the trabeculae superficially. At the same time, no changes in the matrix integrity possibly attributed to the HHP-treatment could be detected.

### Histological Analysis of Trabecular Bone After HHP-Treatment

**FIGURE 9:**
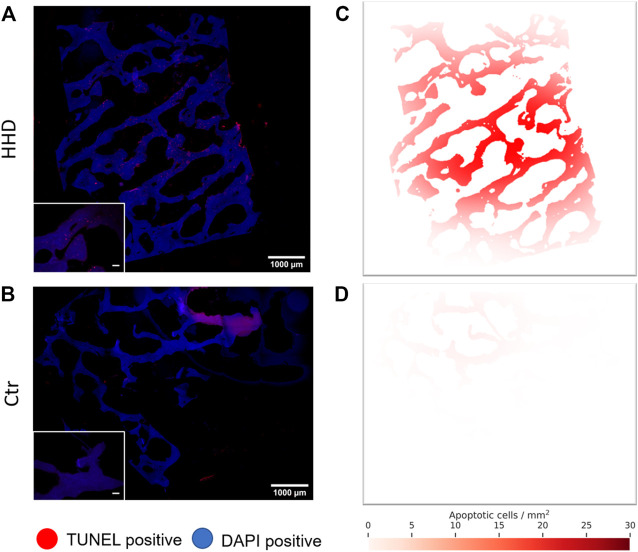
Representative histological fluorescence images of trabecular bone apoptosis, identified via TUNEL Assay. In **(A)** HHD treated sample (300 mPa 20 Min) and **(B)** in an untreated control. Images in the lower left represent magnified sections of the whole sample image, scale bars in these images represent 100 μm. Respective apoptotic cell density maps of the same **(C)** HHD treated sample and **(D)** untreated control were derived from the whole sample image. All images stem from a single donor.

To support our findings regarding the influence of HHD treatment on trabecular bone tissue apoptosis, we created a stochastic model that displays apoptotic cell density correlated to its location in the tissue when applied onto our image data. The performed analysis visualizes qualitative information about the spatial distribution of formerly observed increased apoptosis rates. We were able to show a higher apoptosis rate in HHD treated tissue (Figure 9A) compared to the untreated control throughout the whole sample (Figure 9B) in the images. Additionally, in HHD treated bone tissue, the apoptotic cell density is highest in the center of the sample and decreases gradually towards the outer sample edges (Figure 9C). This was true for all HHD treatment variants, whereas this was not observed in the untreated control group (Figure 9D).

## Discussion

Bone grafting procedures are the second most common tissue transplantations right after blood transfusions (Campana et al., 2014; Wang and Yeung, 2017). Autologous material is still used as the gold standard due to its advantageous bone-building properties. Nevertheless, donor site morbidities can occur and the graft material is limited in terms of removal (García-Gareta et al., 2015). Allogenic or synthetic materials are not limited in availability but they cannot mimic all the positive properties of autologous material to the same extent (García-Gareta et al., 2015). Existing processing methods of allografts often use strong acids/bases, high temperatures or gamma irradiation, which result not only in decellularization and sterilization but also in a reduction of biomechanical properties [12, 28, 29].

High hydrostatic pressure (HHP) represents an alternative to these methods as it has already been shown that at least biomechanical features of bone specimens were not influenced negatively by HHP (Waletzko‐hellwig et al., 2021). Two possible reasons for the preservation of the biomechanics were already discussed in our previous study (Waletzko‐hellwig et al., 2021). Mechanical properties can only be preserved if the extracellular matrix remains intact, structure proteins such as collagen-1 have to be intact and thus retain their supportive function (Viguet-Carrin et al., 2006; Clarke, 2008). FESEM analysis showed no apparent damage to the ECM after HHP-treatment. Apart from some cellular remnants, which are typical of bone cells extending across the matrix, no differences in the ultrastructure of treated samples compared to the control could be detected. In other studies, which investigated thermal and irradiation influences, dose and temperature dependent changes in bone specimens could be observed (Holden et al., 1995; Moreau et al., 2000; Lambri et al., 2016; Rahman et al., 2020). Besides FESEM analysis, those and other groups focused on the structural integrity of proteins, as in the study at hand. Comparing the separation scheme of the proteins of HHP-treated and HHP-untreated samples, distinctive bands could be detected in both cases, which were identified as alpha-1 and alpha-2 chains. The existence and structural integrity of those bands are important for the formation of collagen-1 fibrils, which in turn positively influence mechanical integrity due to their arrangement (Pendleton et al., 2019). In contrast to HHP-treatment, both, thermal and irradiation processing of bone specimens, led to protein degradation. However, a correct arrangement of collagen fibrils is no longer ensured and specimens are mechanically unstable [14, 35, 37,38]. In order to explain why HHP treatment does not affect protein integrity, mechanisms of action of the different processing methods need to be discussed. The temperatures of 1,000°C and more used in industry for thermal processing of allografts lead to an irreversible denaturation of proteins due to polypeptide chain break up (Lambri et al., 2016; Botiss-dental, 2021). Gamma irradiation causes either polypeptide chain scissions due to direct ionization or changes in crosslinking through the formation of highly reactive hydroxyl radicals, which both lead to a loss of protein integrity (Sloff et al., 2018; Silva Aquino, 2012). In both cases, the primary structure of proteins is destroyed. Proteins exposed to HHP follow the principle of Le Châtelier. If a pressure is applied to a chemical equilibrium, the equilibrium shifts to the state which requires the least volume (Queirós et al., 2018). This means the proteins are modified in their structure due to the dissolution of cavities or hydrogen bonds which is reversible. Only at pressures higher than 700 MPa, primary and secondary structures are damaged irreversible (Rodiles-López et al., 2010). SDS-PAGE samples were prepared in such a way that the primary structure is maintained. Since HHP-treatment did not impair the primary protein structure but gamma irradiation or thermal processing does as various studies showed, separated bands are detected in the first case and only a smear in the last two cases.

In addition to the preserved protein integrity, it was also shown that resident tissue cells react apoptotic or necrotic to HHP. Necrosis leads to a rupture of cells, releasing intracellular components. This may cause immunogenic reactions in the recipient of the transplanted graft. Apoptosis results in the formation of apoptotic bodies in which intracellular debris is enclosed. Released debris can easily be found and phagocytosed by macrophages which makes an apoptotic decellularization more desirable (Le et al., 2020). Via DNA fragment analysis the specific DNA ladder pattern as a result of selective DNA cleavage to base pairs of approx. 180 bp in size could be detected. In some cases, a necrotic reaction was also observed, which was found in a smear in the DNA gel. This was mainly true for pressures of 300 MPa, but could not be observed for all donors, which in part suggests a sample or donor dependent reaction. It should also be noted that a pressure of 300 MPa is often described as a threshold in the transition from apoptosis to necrosis (Frey et al., 2008; Rivalain et al., 2010). Those particular DNA ladder patterns have already been reported by various groups as a reliable indication of apoptosis and necrosis (Bortner et al., 1995; Zhang and Xu, 2000; Majtnerová and Roušar, 2018). Another independent assay evaluated the DNA fragmentation based on histological sections via TUNEL assay. This method labels the double strand DNA breaks which are generated during apoptosis at the 3′-OH termini. TUNEL is considered to be more sensitive than DNA fragment analysis via gel electrophoresis, as it precedes complete DNA fragmentation in the apoptosis process (Majtnerová and Roušar, 2018). However, it is also advised not to use these methods isolated, as false positive results have also been reported (Loo, 2011; Majtnerová and Roušar, 2018). Since both methods independently conclude an apoptotic effect of HHP in cancellous bone, the presented results can be considered reliable.

Histological analysis of the HHP treated tissue answers the question whether the HHP application is uniformly effective all across the tissue. The highest density of apoptotic cells was noticed in the center of the samples. However, this should not lead to the conclusion that the cells at the outer edge were unaffected by HHP. During apoptosis cell form apoptotic bodies which therefore lose their original structure and attachment to the tissue matrix (Majtnerová and Roušar, 2018). This leads to the assumption that those apoptotic bodies at the outer edge diffuse out of the tissue, while those in the center of the sample stay put.

Gene expression also confirmed the hints regarding the apoptotic behavior of bone-associated cells. To interpret gene expression analysis, the apoptotic signaling pathway should be examined more closely. Apart from a few modifications, apoptosis is basically classified into an extrinsic and an intrinsic pathway, both resulting in the cleavage of DNA (Elmore, 2007). If an external stimulus triggers the extrinsic pathway this results in an attachment of the membranous death receptor ligand (*fas*) of cell A to a membranous death receptor of cell B. This leads to the activation of an intracellular signaling cascade in cell B. With the activation of caspase-8 two signaling ways are possible. On the one hand a direct activation of caspase-3 is feasible. On the other hand, caspase-8 can activate the BH3 interacting-domain death agonist (BID) which is part of the intrinsic pathway. Further signaling then leads to an activation of caspase-9 and results again in an activation of caspase-3 (Elmore, 2007; Yokobori et al., 2014). Since this study showed that both *fas* and *caspase-8* were overexpressed after HHP-treatment it can be suggested that HHP initially leads to the activation of the extrinsic pathway. Regarding gel electrophoresis and histological staining, it can be assumed that the signaling cascade reaches the DNA cleavage step. However, the question of whether caspase-3 is activated directly or via an intermediate route via the intrinsic pathway cannot be answered with the available data.

It is also striking that the period of the selected incubation time after HHP is related to the degree of gene expression of *fas* and *caspase-8*. Globally, both *fas* and *caspase-8* were overexpressed at 0 h after HHP. Short HHP-treatments of 10 min led to a steady decrease of this overexpression over time, longer HHP-treatments of 20 and 30 min led to a fluctuation of gene expression. A possible explanation for this is that the signal of apoptosis is passed on from cell to cell. It should also be noted that apoptosis is a reversible process if, e.g., the stimulus is not sufficient or is removed too early (Elmore, 2007). It is therefore conceivable that a treatment of 10 min is not sufficient to irreversibly induce apoptosis in bone specimens.

Comparing the gene expression data of longer HHP applications with the data of a short HHP-treatment, it can be suggested that the cells in the tissue already activate the signaling cascade during HHP-treatment. This can be demonstrated by the following example. Comparing the expression of *caspase-8* at a pressure of 250 MPa for 30 min at the incubation time 0 h with the expression at a pressure of 250 MPa for 10 min at the incubation time 1 h, gene expressions are nearly at the same level. This also applies to the same pressures and *fas* expression. Therefore, a longer HHP application without subsequent incubation has a comparable effect to a shorter HHP application with subsequent longer incubation. This leads to the assumption that apoptosis signaling pathway in HHP-treated tissue progresses further with a prolonged HHP application.

Like all studies, the one at hand also has its limitations. Although there are various indications that pressures applied here lead to apoptosis, the complete signaling cascade could not be reconstructed using the available methods. Therefore, possible approaches could be further gene expression analysis of BID or caspase-9. A conceivable alternative would also be a specialized live-cell imaging analogous to the work of Schneidereit et al. (2019). Although this chamber can only be used to apply a pressure of 200 MPa (lower than the pressures used in this study), this method could still provide further information on the influence of HHP. An important aspect for later clinical application is the question whether HHP preserves the proteins’ functionality as well as their structural integrity. The unfolding and refolding could lead to a loss in the activity of proteins, however immunologically active surface markers could also be hidden. This question could be answered with an activity assay and a three-dimensional structure analysis via protein crystallization. Despite the limitations mentioned here, this study is reliable due to the different methodological approaches (DNA-/RNA-/protein level and histology) and the selected sample sizes of four and more.

An essential aspect, that was not taken into account in the present study is the remaining immunological potential of the bone grafts. Besides resident proteins, which could possibly lead to graft rejection, increased immune reaction of the recipients to the devitalized cells cannot be ruled out. Although the collected data provide information and indications about the type and distribution of cell death, it is not possible to estimate the exact immune responses from this data alone. Future research should focus on this remaining immunological potential. In order to answer this question *in vitro* studies in which PBMCs are incubated with HHP treated and untreated bone blocks would be conceivable. With this, e.g., the released pro-an anti-inflammatory cytokines could be detected, which could provide information regarding the type of immune response. If the results of these studies are promising, more comprehensive *in vivo* studies could follow to give an overview of the reaction to the transplant, locally in the peri-implant tissue, as well as systemically, in the whole organism.

Another point of interest which has not been considered here is residual microbiota. Like different cell types, various bacteria and virus strains react differently to HHP. Investigations showed a complete inactivation e.g., of *Staphylococcus aureus* but this required a pressure of 600 MPa (Gollwitzer et al., 2009). At this pressure, however, it can be assumed that bone associated cells will react necrotically. Therefore, an immune reaction cannot be excluded. In studies focusing on the residual bacterial and viral load of HHP-treated allografts, it should be investigated which strains are to be expected in a bone allograft and whether lower pressures might also be sufficient to inactivate the microbiota. In order to assess the reaction of other cells to the immune response or the differentiation capacity of the HHP processed allografts, revitalization experiments with monocytes, macrophages or mesenchymal stem cells could be an option.

In conclusion, HHP-treatment has a devitalizing effect which is a gentle alternative compared to other applications. For further application, pressure levels and durations should be chosen carefully as a too short treatment may not lead to complete devitalization and too high pressure may lead to a necrotic reaction which among other aspects, could lead to a strong immune reaction. Based on this study, a reasonable application would be 250 MPa for 20 min. On the one hand, apoptosis is presumably induced to such an extent that the signaling cascade is not interrupted. On the other hand, the probability of necrosis is lower than at 300 MPa. In order to be able to discuss HHP treatment as an alternative to already existing methods of preparing bone substitute materials, further *in vitro* and *in vivo* studies regarding immunological reactions must be carried out. Based on this, revitalization of HHP treated materials with e.g., stem cells could be performed to determine the differentiation capacity and therefore the osteogenic and osteoinductive potential of bone grafts.

## Data Availability

The raw data supporting the conclusion of this article will be made available by the authors, without undue reservation.
